# Personality, usage, and perceptions of AI in medical education: evidence from senior pre-clinical students in China

**DOI:** 10.3389/fpsyg.2026.1805800

**Published:** 2026-03-13

**Authors:** Rixiang Xu, Chengyang Hu, Tingyu Mu

**Affiliations:** 1 School of Medical Humanities, Anhui Medical University, Hefei, China; 2 School of Nursing, Anhui Medical University, Hefei, China

**Keywords:** AI, clinical students, medical education, personality traits, technology adoption

## Abstract

**Background:**

As artificial intelligence (AI) tools rapidly permeate medical education, understanding individual differences in their adoption becomes increasingly important. Personality traits, particularly those defined by the Five-Factor Model, may influence how students engage with AI for learning and how they perceive its value.

**Objective:**

This study aimed to examine the associations between personality traits and both AI tool use behaviors and attitudes among fourth-year clinical medical students in China.

**Methods:**

A cross-sectional survey was conducted among 661 fourth-year clinical students at a medical university in Anhui Province. Personality traits were assessed using the Ten-Item Personality Inventory (TIPI). AI use frequency, specific AI-assisted learning behaviors, and attitudes toward AI in medical education were measured via structured questionnaires. Ordinal and binary logistic regressions were used to analyze behavioral outcomes, while multiple linear regression examined attitudinal associations.

**Results:**

Neuroticism was negatively associated with overall AI use frequency (OR = 0.90, 95% CI: 0.83–0.98). Openness was positively linked to using AI for literature translation (OR = 1.14, 95% CI: 1.04–1.245), and conscientiousness predicted use for Auxiliary examination learning (OR = 1.15, 95% CI: 1.045–1.26). Conscientiousness, agreeableness, and openness were significantly associated with more positive attitudes toward AI's educational and clinical utility. Neuroticism was associated with greater concern over data privacy.

**Conclusions:**

Personality traits meaningfully shape how students interact with AI tools and perceive their role in medical training. Tailored AI literacy programs and supportive learning environments may improve equitable adoption and optimize educational outcomes.

## 1 Introduction

The rapid emergence of artificial intelligence (AI) tools is reshaping medical education worldwide (Sami et al., 2025). Recent advances in large language models, such as ChatGPT, have demonstrated the capacity to perform at or near the level of medical trainees on standardized examinations and clinical reasoning tasks (Maaß et al., 2025; Nagi et al., 2023). In educational settings, AI tools are increasingly used to support knowledge acquisition, clinical case simulation, literature analysis, and communication training, offering scalable and personalized learning support (Lee, 2024; Boscardin et al., 2024). In China, the integration of AI into medical education has accelerated alongside broader digital health initiatives. Surveys indicate that AI-assisted learning tools are widely used by Chinese medical students, with high reported frequency and engagement across multiple learning tasks (Shi et al., 2026). These developments highlight AI's growing role in medical training while raising important questions about how students interact with and perceive such technologies.

Technology adoption in education, however, is not uniform across learners. Beyond institutional availability and perceived usefulness, individual differences play a critical role in shaping how students engage with emerging digital tools. Personality traits, commonly conceptualized within the Big Five framework, have been shown to influence learning strategies, technology use, and risk perception across educational and professional contexts (Arpaci and Kocadag Unver, 2020; Arpaci et al., 2025). Openness to experience is associated with curiosity and willingness to explore novel tools, while conscientiousness reflects goal-oriented and structured learning behaviors. Extraversion and agreeableness relate to interpersonal orientation and may shape views on AI-mediated communication, whereas neuroticism may heighten sensitivity to uncertainty, ethical concerns, and perceived risks (Arpaci et al., 2025). Despite their theoretical relevance, personality traits have rarely been examined in relation to AI use in medical education, particularly with respect to both behavioral engagement and attitudinal evaluation.

Existing empirical evidence remains limited. Most studies on AI in medical education focus on overall acceptance, perceived benefits, or ethical concerns, without accounting for personality-based heterogeneity (Weidener and Fischer, 2023). A recent multicenter study in China reported that openness and agreeableness were associated with more positive attitudes toward AI, whereas conscientiousness showed an inverse association (Qi et al., 2026). However, that study did not examine specific AI use behaviors, nor did it focus on students approaching clinical training. Fourth-year clinical medical students represent a particularly relevant group, as they have completed most foundational coursework and are transitioning toward clinical practice (Zhang et al., 2022), where AI tools may increasingly influence learning, decision-making, and professional development. Understanding how personality traits shape AI use and perceptions at this stage may provide valuable insights for curriculum design and educational policy.

Accordingly, this study aims to examine the associations between personality traits and both AI tool use behaviors and AI-related attitudes among fourth-year clinical medical students in a Chinese medical school. Personality traits were assessed using the Ten-Item Personality Inventory (TIPI), and AI engagement was evaluated across multiple functional domains, including learning support, clinical simulation, and academic tasks. Attitudinal measures captured perceptions of AI's impact on medical education, healthcare quality, professional competencies, specialty choice, humanistic care, and data privacy. By integrating behavioral and attitudinal outcomes, this study seeks to clarify how stable personality characteristics shape students' engagement with AI technologies. The findings are intended to inform more personalized and ethically grounded approaches to integrating AI into medical education, recognizing learner diversity while supporting the responsible development of future physicians.

## 2 Materials and methods

### 2.1 Study design and participants

This cross-sectional study was conducted among fourth-year undergraduate students enrolled in a 5-year clinical medicine program at a medical university in Anhui Province, China. Fourth-year students were selected because they had completed most core theoretical medical courses and were preparing to enter formal clinical rotations, representing a critical transitional stage in medical training. Participants were recruited using a cluster sampling strategy. Several classes were randomly selected from the eligible cohort, and all students within the selected classes were invited to participate. The inclusion criteria were enrollment in the 5-year clinical medicine undergraduate program and current registration in the fourth academic year. Students from other majors or academic years were excluded. A total of 721 questionnaires were distributed, and 661 valid responses were received, yielding a response rate of 91.8%. Data were collected between November 1 and December 15, 2025. All participants provided informed consent and completed the survey anonymously.

### 2.2 Data collection procedure

Data were collected using a self-administered, anonymous online questionnaire. The survey link was distributed to students through class-based communication channels. Participation was voluntary, and informed consent was obtained electronically before students accessed the questionnaire. To minimize response bias, students were informed that the survey was for research purposes only and that their responses would not affect academic evaluation.

### 2.3 Measures

#### 2.3.1 Personality traits

Personality traits were assessed using the Ten-Item TIPI, a brief and widely used instrument designed to measure the Big Five personality dimensions: extraversion, agreeableness, conscientiousness, neuroticism, and openness to experience (Shi et al., 2022). Each personality dimension is assessed using two items, resulting in a total of 10 items. All items were rated on a seven-point Likert scale ranging from “strongly disagree” to “strongly agree.” Scores for each personality trait were calculated following standard scoring procedures reported in previous validation studies, with higher scores indicating a stronger expression of the corresponding trait (Ardenghi et al., 2024).

#### 2.3.2 AI Tool use behaviors

AI tool use was assessed from two perspectives: overall frequency of use and engagement in specific functional applications. Overall frequency of AI tool use was measured as an ordinal variable reflecting how often students used AI tools for learning-related purposes. Specific AI use behaviors were measured as binary variables (yes/no), indicating whether students had used AI tools for the following purposes: assisting comprehension of complex medical concepts; generating study outlines or knowledge summaries; generating self-assessment exercises; translating medical literature; generating or analyzing clinical case scenarios; simulating patient–physician dialogues; assisting learning of auxiliary examinations such as medical imaging or electrocardiograms; assisting in writing case reports; and assisting in completing coursework or academic reports.

#### 2.3.3 Attitudes toward AI

Students' attitudes toward AI were assessed using multiple items capturing perceptions of AI's impact on medical education and future medical practice. Perceived overall impact of AI on medical education was measured using a bipolar scale ranging from −5 (“absolute harm”) to +5 (“absolute benefit”), allowing respondents to indicate whether they viewed AI as predominantly beneficial or detrimental to medical education. Other attitude items, including perceived enhancement of healthcare quality and safety, perceived importance of AI skills as a core physician competency, perceived influence of AI on future subspecialty choice, concerns about potential decline in humanistic care, and concerns regarding data privacy and security risks, were measured using five-point Likert scales ranging from 1 (“strongly disagree”) to 5 (“strongly agree”). Higher scores indicated stronger endorsement of the corresponding perception or concern.

#### 2.3.4 Covariates

Several covariates were included to control for potential confounding effects. This included gender, age, place of residence, plan to pursue further education, only-child status, and prior exposure to AI-related training.

### 2.4 Statistical analysis

Descriptive statistics were used to summarize participant characteristics and distributions of key variables. Continuous variables were reported as means and standard deviations, and categorical variables were summarized using frequencies and percentages. Associations between personality traits and AI tool use frequency were examined using ordinal logistic regression models, given the ordered nature of the frequency variable. Associations between personality traits and specific AI use behaviors were analyzed using binary logistic regression models, with each AI application treated as a dependent variable. Associations between personality traits and AI-related attitudes were examined using multiple linear regression models. In all regression analyses, personality traits were entered as independent variables. Regression results were reported as odds ratios (ORs) with 95% confidence intervals (CIs) for logistic models and as unstandardized regression coefficients (*B*) with 95% CIs for linear models. Statistical significance was determined based on whether the 95% CI excluded the null value. All analyses were conducted using standard statistical software. A two-sided *p* value < 0.05 was considered statistically significant.

## 3 Results

### 3.1 Participant characteristics

Descriptive statistics for personality traits, AI tool use behaviors, and AI-related attitudes are presented in Table 1. The mean age of participants was 21.14 years (SD = 0.79), and 57.79% (382/661) were male. Most students reported an urban place of residence (69.74%, 461/661), and 35.70% (236/661) were only children. The majority of participants reported plans to pursue further education (97.28%, 643/661), while a small proportion reported no such plans (0.61%, 4/661) or were undecided (2.12%, 14/661). Prior AI-related training was reported by 41.90% (277/661) of students. Among personality dimensions measured by the Ten-Item Personality Inventory, openness to experience showed the highest mean score (mean = 9.66, SD = 2.02), followed by conscientiousness (mean = 9.13, SD = 2.01) and agreeableness (mean = 9.03, SD = 1.66).

**Table 1:** Participant characteristics and descriptive statistics (*N* = 661).

**Characteristic**	***n* (%) or Mean (SD)**
**Age**, years	21.14 ± 0.79
**Gender**	
Male	382 (57.79)
Female	279 (42.21)
**Place of residence**	
Urban	461 (69.74)
Rural	200 (30.26)
**Only-child status**	
Yes	236 (35.7)
No	425 (64.3)
**Plan to pursue further education**	
Yes	643 (97.28)
No	4 (0.61)
Undecided	14 (2.12)
**Prior AI-related training**	
Yes	277 (41.9)
No	384 (58.1)
**Personality traits**	
TIPI-E	7.93 ± 2.08
TIPI-A	9.03 ± 1.66
TIPI-C	9.13 ± 2.01
TIPI-N	7.14 ± 2.00
TIPI-OE	9.66 ± 2.02
**Frequency of AI tool use**	
Almost daily	183 (27.7)
Several times per week	291 (44.0)
Several times per month	140 (21.2)
Rarely	40 (6.1)
Never	7 (1.1)

TIPI-E, Extraversion; TIPI-A, Agreeableness; TIPI-C, Conscientiousness; TIPI-N, Neuroticism; TIPI-OE, Openness to Experience.

### 3.2 Patterns of AI tool use and attitudes

**Figure 1:**
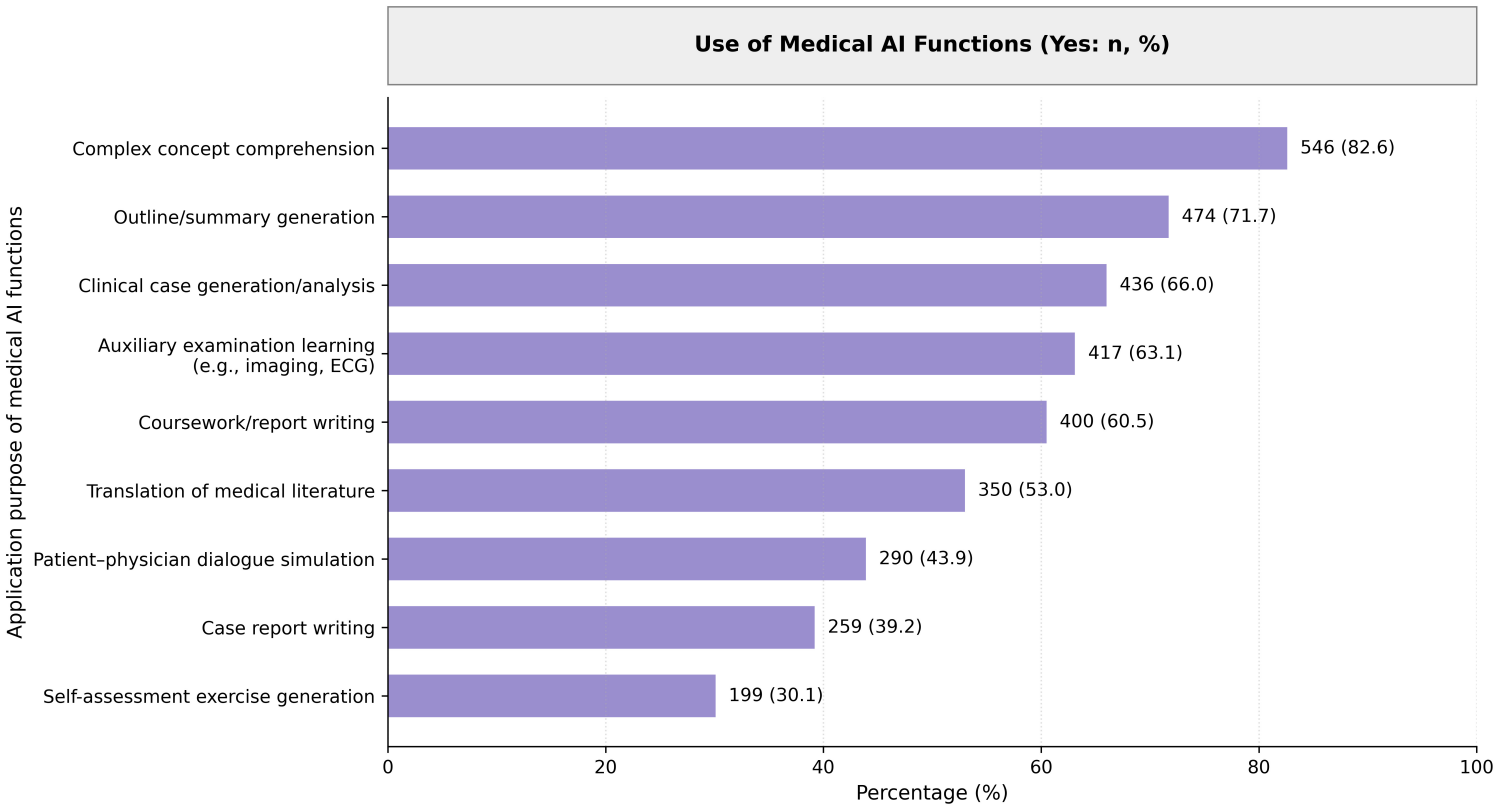
Purpose of AI tool use among fourth-year clinical medical students.

AI tool use was widespread among participants, with 71.70% reporting use at least several times per week. Most students used AI for tasks such as complex concept comprehension (82.60%), summary generation (71.70%), and clinical case analysis (66.00%). Other common applications included interpreting auxiliary tests (63.10%), coursework writing (60.50%), and literature translation (53.00%). Less frequent uses included patient–physician dialogue simulation (43.90%) and self-assessment generation (30.10%). Full usage patterns are illustrated in Figure 1.

**Figure 2:**
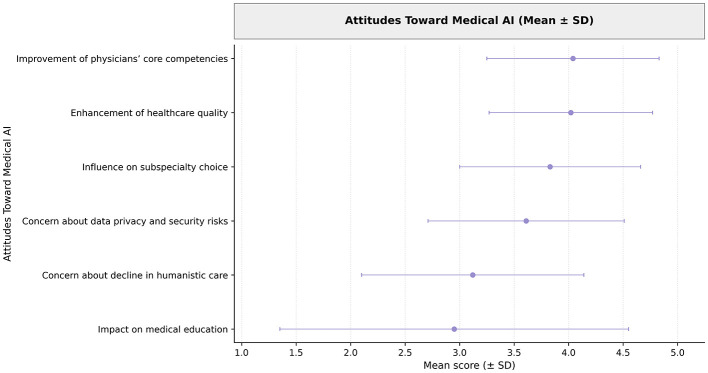
Attitudinal scores toward AI among fourth-year clinical medical students.

Overall attitudes toward AI were shown in Figure 2. Students strongly agreed that AI could improve healthcare quality (mean = 4.02, SD = 0.75) and physicians' core competencies (mean = 4.04, SD = 0.79). They also viewed AI as a moderate positive force in medical education (mean = 2.95 on a −5 to +5 scale). Concerns about data privacy (mean = 3.61) were more prevalent than concerns about a decline in humanistic care (mean = 3.12). These trends reflect both optimism and caution in students' perspectives on AI integration in medical learning.

### 3.3 Associations between personality traits and AI tool use frequency

**Figure 3:**
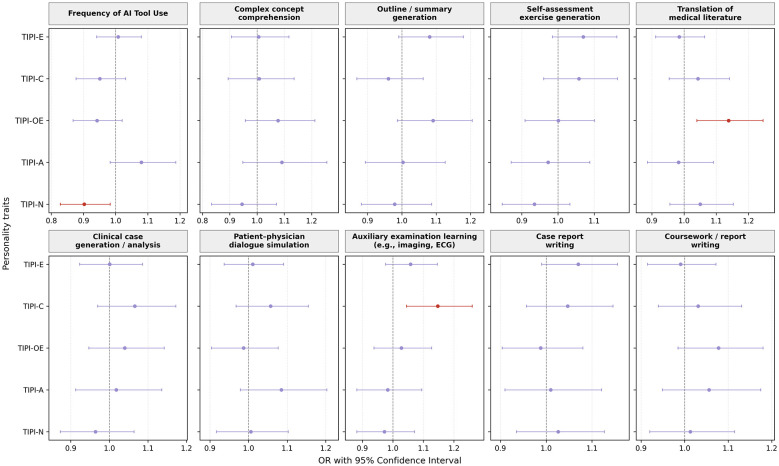
Associations between personality traits and AI use frequency and specific AI-assisted learning behaviors. TIPI-E, Extraversion; TIPI-A, Agreeableness; TIPI-C, Conscientiousness; TIPI-N, Neuroticism; TIPI-OE, Openness to Experience.

Ordinal logistic regression analysis was conducted to examine the association between personality traits and overall frequency of AI tool use (Figure 3). After adjusting for covariates, neuroticism was significantly associated with AI use frequency. Higher levels of neuroticism were associated with a lower frequency of AI tool use (OR = 0.90, 95% CI: 0.83–0.98). No statistically significant associations were observed between AI use frequency and extraversion, conscientiousness, openness to experience, or agreeableness.

### 3.4 Associations between personality traits and specific AI use behaviors

Binary logistic regression models were used to assess associations between personality traits and engagement in specific AI-assisted learning behaviors (Figure 3). Openness to experience was positively associated with the use of AI tools for translating medical literature (OR = 1.14, 95% CI: 1.04–1.25). Conscientiousness was positively associated with the use of AI tools for generating or analyzing clinical case scenarios (OR = 1.15, 95% CI: 1.05–1.26). No other statistically significant associations were identified between personality traits and specific AI use behaviors, including concept comprehension, outline or summary generation, self-assessment exercise generation, patient–physician dialogue simulation, auxiliary examination learning, case report writing, or coursework and report writing.

### 3.5 Associations between personality traits and attitudes toward AI

**Figure 4:**
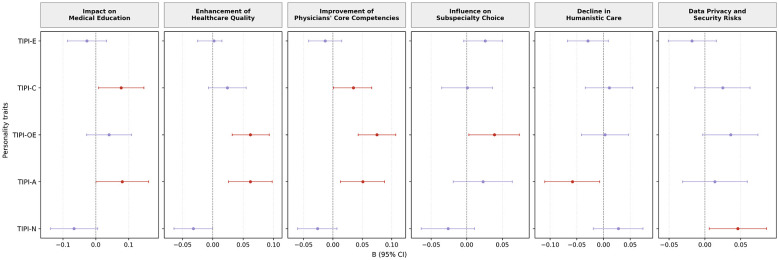
Associations between personality traits and attitudes toward AI in medical education and future practice. TIPI-E, Extraversion; TIPI-A, Agreeableness; TIPI-C, Conscientiousness; TIPI-N, Neuroticism; TIPI-OE, Openness to Experience.

Multiple linear regression analyses were conducted to examine the associations between personality traits and AI-related attitudes (Figure 4). Conscientiousness and agreeableness were both positively associated with perceived overall impact of AI on medical education (*B* = 0.77, 95% CI: 0.09–1.46; *B* = 0.81, 95% CI: 0.00–1.61, respectively). Openness to experience and agreeableness were positively associated with perceived enhancement of healthcare quality and safety. In addition, conscientiousness, openness to experience, and agreeableness were all positively associated with perceived improvement of physicians' core competencies. Openness to experience was positively associated with perceived influence of AI on future subspecialty choice (*B* = 0.04, 95% CI: 0.00–0.07). Agreeableness was negatively associated with concerns regarding a decline in humanistic care (*B* = −0.06, 95% CI: −0.11 to −0.01). Neuroticism was positively associated with concerns related to data privacy and security risks (*B* = 0.05, 95% CI: 0.01–0.09). No statistically significant associations were observed for extraversion across attitude domains.

## 4 Discussion

This study reveals that distinct personality traits are meaningfully associated with both the frequency and pattern of AI tool use as well as students' attitudes toward these technologies in clinical medical education. Specifically, higher levels of neuroticism were linked to less frequent engagement with AI tools, while traits such as conscientiousness and openness to experience were associated with more targeted usage, such as applying AI for clinical case analysis or literature translation. In terms of attitudes, students scoring higher on conscientiousness, openness, and agreeableness generally expressed more favorable views of AI's educational value, clinical potential, and future relevance. Conversely, neuroticism was associated with increased concerns about data privacy and technological risks. Together, these findings suggest that Big Five personality dimensions play an important role in shaping how students engage with and interpret the role of AI in their learning.

Our results indicate that individual differences shape how students adopt and perceive AI in medical training. The negative relationship between neuroticism and AI use frequency aligns with evidence that highly neurotic individuals often find new technologies stressful and tend to avoid them (Arpaci et al., 2025; Devaraj et al., 2008). In our study, anxious or emotionally unstable students were indeed less inclined to use AI regularly. They also voiced more concern about data privacy, consistent with findings that neuroticism correlates with heightened sensitivity to privacy risks (Bansal et al., 2010). These students may be wary of AI, highlighting the need for support and training. In contrast, openness to experience and conscientiousness appear to facilitate greater engagement with AI. Open individuals–curious and eager to explore–are known to adopt new technologies more readily (Ozbey and Yasa, 2025; Park and Woo, 2022). Accordingly, open students in our sample were more likely to use AI for tasks like translating literature, likely to overcome language barriers in accessing research. Conscientious students, who are disciplined and achievement-oriented, similarly embraced AI as a learning aid. They used AI for clinical case analysis, reflecting a strategic approach to bolster their understanding. Conscientious people tend to adopt tools if they perceive a performance benefit (Major et al., 2006), so these students likely saw AI as helpful for mastering clinical knowledge. Notably, conscientious students also held optimistic views of AI's educational value, indicating they see AI as aligning with their goals of efficiency and competence.

Agreeableness was linked to broadly positive attitudes toward AI. This trait, associated with trust and cooperativeness, has been shown to predict favorable views of AI technology (Stein et al., 2024). In our context, agreeable students were enthusiastic about AI's benefits (for education, healthcare quality, and physician skills) and relatively unconcerned by fears of dehumanization. Being trusting, they may view AI as a collaborative tool rather than a threat. Their lower worry that AI could undermine humanistic care suggests an assumption that empathy and technology can coexist—a perspective contrasting with common concerns about AI depersonalizing healthcare (Akingbola et al., 2024).

These personality-driven tendencies play out against the backdrop of China's medical education system. The curriculum's heavy emphasis on content mastery and high-stakes exams often overshadows opportunities for technology integration (Tong et al., 2024). Indeed, a recent national survey found that Chinese medical students have moderate knowledge and attitudes about AI but low actual usage, highlighting an AI literacy gap (Li et al., 2026). In this environment, only the more self-motivated or tech-curious students (typically those high in openness or conscientiousness) may proactively use AI tools, while others lag behind. Furthermore, institutional policies shape attitudes: for example, some universities have restricted use of generative AI like ChatGPT due to concerns about cheating or data security (Wang et al., 2025). Such restrictions, while addressing real risks, might reinforce apprehension in students who are already hesitant (such as those high in neuroticism). Therefore, beyond considering personality factors alone, it is crucial to create a supportive educational environment that offers guidance on responsible AI use. Rather than relying solely on prohibitions, such an approach ensures that all students have the opportunity to benefit from AI innovations.

To harness AI's potential in medical education while addressing the diverse needs of learners, we propose a multifaceted approach. First, integrating AI literacy training into the curriculum is essential. Practical modules should provide students with hands-on experience using AI through activities such as clinical simulations, data analysis, and literature review. This exposure can help bridge the gap between students' moderate awareness of AI and their relatively low levels of actual usage (Li et al., 2026). Second, clear ethical use guidelines must be established to define acceptable applications of AI in coursework and assessments (Tam et al., 2023). This includes distinguishing between legitimate assistance, such as using AI for research or translation, and misuse, such as having AI complete entire assignments. Transparency in AI use should be encouraged, supported by honor codes, citation protocols, and plagiarism detection measures. Third, AI-related instruction should be adapted to students' personality profiles. Open-minded students may thrive in exploratory environments, while those with high conscientiousness or neuroticism may benefit from structured guidance and reassurance about AI reliability. Second, educational content should proactively address student concerns. Finally, peer learning can be leveraged. Extraverted and tech-confident students could serve as informal mentors or lead group-based AI learning activities. Pairing students with different personality profiles in collaborative exercises could also promote mutual understanding and skill exchange.

These findings underscore the importance of integrating psychological diversity into the ethical and pedagogical design of AI education. By recognizing that traits like openness and neuroticism influence how students engage with AI, educators can tailor support systems accordingly. Institutions should avoid one-size-fits-all approaches and instead foster AI literacy through inclusive strategies that address both technological confidence and ethical concerns. As prior behavioral models suggest, individual and contextual moderators can shape technology adoption trajectories (Mishra et al., 2024; Singh et al., 2024). Integrating personality-aware interventions may promote equitable access and responsible AI integration in medical training.

Several limitations should be noted. This study is cross-sectional, capturing associations at a single point in time and not establishing causality. All measures (including AI use and attitudes) were self-reported, introducing potential bias. The sample may not represent all Chinese medical students (e.g., those from other regions or school types), limiting generalizability. Future research would benefit from longitudinal studies to see how personality and AI use evolve together, and from including objective usage data or broader samples to validate these findings. Future research should incorporate longitudinal designs to examine how personality traits and AI-related behaviors evolve over time. Additionally, exploring the mediating or moderating roles of institutional culture, perceived fairness, or digital content engagement could help uncover deeper mechanisms of AI adoption in education (Agrawal et al., 2025).

## 5 Conclusion

This study provides evidence that medical students' personality traits influence both their AI tool usage and attitudes. Educational strategies that account for individual differences can promote more equitable and effective integration of AI in clinical training. As AI becomes increasingly embedded in medical education and practice, aligning technological tools with learner diversity will be essential for preparing future physicians who are confident, competent, and critical users of AI.

## Data Availability

The original contributions presented in the study are included in the article/supplementary material, further inquiries can be directed to the corresponding authors.
